# Interfacial Engineering of Clay-Based Nanohybrids with pH-Responsive Network-like Behavior for Hair Photoprotection and Algal Growth Promotion

**DOI:** 10.3390/gels12060530

**Published:** 2026-06-12

**Authors:** Hao Chen, Yufan Song

**Affiliations:** School of Pharmaceutical and Chemical Engineering, Taizhou University, Shifu Road No. 1139, Taizhou 318000, China

**Keywords:** nanohybrids, surface modification, colloidal stability, biointerface, UV protection, environmental safety

## Abstract

The interfacial behavior of hybrid nanoparticles on biological substrates governs their functional performance. Here, we investigate how surface properties and colloidal stability dictate the pH-dependent adhesion of oxybenzone-loaded palygorskite nanohybrids to hair—a model biological interface. A series of hybrids with 5–50% oxybenzone loadings were prepared via melt impregnation. XRD and FTIR analyses confirm hydrogen bonding between oxybenzone and palygorskite, forming stable organic–inorganic hybrids. The colloidal stability of these nanohybrids varies non-monotonically with oxybenzone loading, governed by surface hydrophilicity and zeta potential, exhibiting a network-like behavior upon pH change. Optimal stability is achieved at an intermediate loading with a favorable balance of surface properties. While pristine hybrids show no affinity for hair, surface modification with cationic polyquaternium-7 (PQ-7) or non-ionic polyvinylpyrrolidone (PVP) enables effective deposition through distinct pH-dependent mechanisms: PQ-7 operates optimally at pH 10 via electrostatic attraction, whereas PVP performs best at pH 4 through hydrogen bonding, forming a protective coating layer on the hair surface. Deposition fails for PVP-modified hybrids at 50% loading due to excessive surface hydrophobicity. The deposited hybrids provide exceptional UV protection, significantly mitigating cuticle damage, suppressing photo-yellowing, and minimizing protein oxidation. Among the hybrids, hybrid-35 exhibited the best colloidal stability, whereas PQ-7-modified hybrid-50 gave the highest UV protection (color difference Δ*E* reduced from 10.51 to 1.60). The adhesion rates of the two best-performing hybrids were 2.70% and 2.85%, respectively. Beyond hair protection, we evaluate the environmental interface of these materials. While free oxybenzone is highly toxic to *Chlorella vulgaris*, hybridization drastically reduces its ecotoxicity. Remarkably, palygorskite and the hybrids promote algal growth, likely by acting as nutrient adsorbents and attachment sites. This work provides fundamental insights into particle–biointerface interactions and offers a strategy for designing functional hybrid materials with tailored surface properties for bio-related applications.

## 1. Introduction

The development of multifunctional nanomaterials for personal care and biomedical applications has attracted increasing attention due to their ability to integrate multiple functions into a single platform [1,2]. Among these, organic–inorganic hybrid materials, which combine the advantages of organic functional molecules and inorganic carriers, offer great promise for creating advanced materials with tailored surface properties and interfacial interactions [3,4]. Oxybenzone, also known as 2-hydroxy-4-methoxybenzophenone, benzophenone-3, or UV filter UV-9, has the chemical formula C_14_H_12_O_3_. Its extremely low water solubility allows it to form an effective, water- and sweat-resistant film in sunscreen formulations, making it one of the most widely used organic UV filters [3]. Studies have shown that oxybenzone not only induces deformation and mortality in coral larvae but can also be metabolized by organisms such as corals into phototoxic byproducts, directly contributing to coral bleaching, which has been an urgent environmental concern [4,5].

To mitigate the environmental impact of sunscreen agents while maintaining their performance, current research follows two main directions: the development of novel eco-friendly alternative substances, and the application of materials science approaches to encapsulate existing UV filters [6]. In the search for alternatives, research has concentrated on exploring the photoprotective potential of natural plant extracts, such as those from red onion and dragon fruit peel [7,8]. Concerning material-based encapsulation, constructing organic–inorganic hybrid materials to reduce the bioavailability and environmental mobility of UV filters is considered a highly promising route [9,10]. This strategy involves encapsulating organic sunscreen molecules within porous inorganic carriers, such as zeolites or mesoporous silica [11,12]. This approach retains the ultraviolet absorption functionality while reducing dissolution and release into aqueous environments through physical barrier effects, thereby lowering the environmental burden at the source [13]. Simultaneously, the inorganic matrix can isolate sunscreen molecules, inhibit photodegradation, and consequently enhance the photostability and long-lasting protective efficacy of the product [14]. This strategy of engineering hybrid materials with controlled surface properties and interfacial interactions is central to the design of advanced functional hybrid materials.

Clay minerals, being low-cost, abundant, and exhibiting excellent biocompatibility, represent highly competitive candidate materials as carriers for sunscreen molecules [15]. Some clay minerals even possess inherent ultraviolet shielding properties [16]. To date, successful preparations of inorganic–inorganic composite sunscreen materials based on clay minerals, such as TiO_2_ [13], ZnO [17], and CeO_2_ [18], have been reported, demonstrating enhanced UV protection and stability. For organic sunscreens, research has primarily focused on layered clay minerals like montmorillonite and layered double hydroxides (LDHs). The strategy involves intercalating sunscreen molecules into the interlayer spaces of the clays, which has been shown to improve photostability, safety, and sunscreen performance [19,20,21,22,23]. Among these, only two studies specifically involve oxybenzone [22,23]. Reports on the combination of other structural clay minerals with sunscreen molecules are scarce [24]. Furthermore, the aforementioned studies have concentrated on evaluating the materials’ sunscreen performance, with no investigation into their impact on aquatic organisms or their interactions with biological substrates such as hair.

Palygorskite, a magnesium-rich fibrous nanoclay mineral, possesses a unique one-dimensional nanorod-like crystal structure and a regular nano-channel system [25,26]. Individual rod crystals have diameters of approximately 20–70 nm and lengths ranging from 0.5 to 5 μm, making it a naturally occurring one-dimensional nanomaterial with high aspect ratio. These characteristics endow palygorskite with excellent ion exchange capacity, adsorption properties, and thermal stability, leading to its wide application in areas such as catalysis, environmental adsorption, drug delivery, and composite reinforcement [27,28]. The abundant surface hydroxyl groups on palygorskite also provide versatile sites for surface functionalization, enabling the tuning of its surface charge, hydrophilicity, and colloidal behavior. However, its application in sunscreen-related research is currently limited. Only one study by Liu et al. reported a CeO_2_/palygorskite/lignin composite fabricated via a biomimetic strategy, mimicking the multi-level barrier and antioxidant mechanisms of plant leaves to achieve synergistic UV shielding [24].

Although people generally prioritize sun protection for the face and exposed skin, hair is often overlooked. Hair acts as an important barrier protecting the scalp. Its internal melanin effectively absorbs UV radiation, while the keratin structure maintains the strength and elasticity of hair strands. However, UV radiation can degrade melanin, leading to “photo-yellowing,” and oxidize keratin, disrupting disulfide bonds. This results in cuticle damage, increased hair fragility, and ultimately compromises its photoprotective function for the scalp [29]. Therefore, developing effective hair sunscreens is crucial for maintaining its physiological protective function and structural integrity. To date, there is only one report on the application of clay minerals for hair sun protection: Cavallaro et al. designed and prepared a halloysite/keratin nanocomposite. By forming a coating on the hair surface, it significantly blocked UV-induced photochemical damage, offering a novel material design concept and a feasible technical pathway for developing new, efficient, and environmentally friendly hair care products [30]. However, the fundamental relationships between particle surface properties, colloidal stability, and adhesion to hair remain poorly understood.

Oxybenzone (BP-3) exhibits good thermal stability [31], while its melting point is only about 62–64 °C, making it feasible to readily prepare uniform BP-3–clay organic–inorganic nanohybrid materials via the melt-impregnation method. The term “hybrid nanoparticle” refers to a structure composed of an inorganic palygorskite nanoclay core with oxybenzone (organic UV filter) loaded onto its surface and into inter-rod spaces via hydrogen bonding and physical entrapment. This differs from conventional nanoparticles (e.g., pure polymer or metal nanoparticles) by combining organic and inorganic components at the nanoscale, providing synergistic properties. Based on these considerations, a series of oxybenzone–palygorskite hybrids with different mass ratios were prepared in this study. The hybrids were further modified with two surfactants, polyquaternium-7 and polyvinylpyrrolidone, to modulate their surface properties and colloidal behavior. The pH-dependent aggregation and dispersion of the hybrids exhibit a network-like behavior. The term “network-like” is used descriptively to refer to the pH-responsive aggregation/dispersion behavior of the hybrids, not to imply true gel rheology. We systematically investigated how these surface modifications influence the adhesion performance of the hybrids on hair—a model biological substrate—and elucidated the underlying sun-protection mechanism. Additionally, the impact of the hybrid materials on the growth of the aquatic microalga *Chlorella vulgaris* was evaluated to assess their environmental compatibility.

The objectives and novelty of this work are threefold: (i) to systematically correlate particle characteristics (size, surface charge, wettability) with colloidal stability and pH-dependent adhesion; (ii) to elucidate two distinct adhesion mechanisms (electrostatic attraction vs. hydrogen bonding) enabled by surface modification with PQ-7 or PVP; and (iii) to demonstrate the dual functionality of the hybrids as effective hair photoprotectors while reducing ecotoxicity and even promoting algal growth.

## 2. Results and Discussion

### 2.1. Microscopic Morphology of Materials

**Figure 1:**
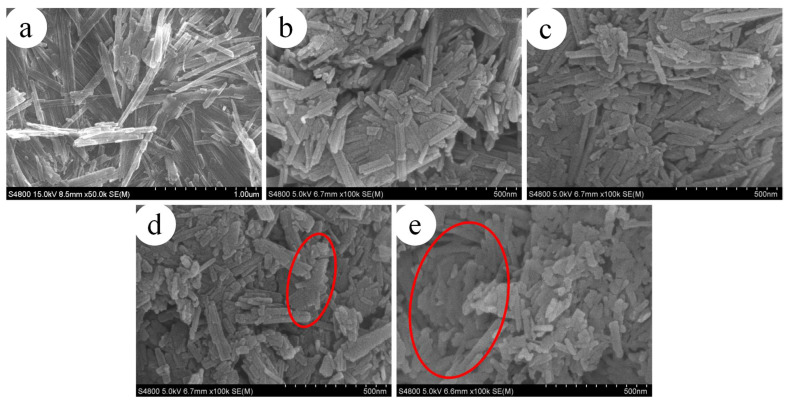
SEM photos of palygorskite (**a**), hybrid-5 (**b**), hybrid-20 (**c**), hybrid-35 (**d**), and hybrid-50 (**e**).

To investigate the effect of oxybenzone loading on the microstructure of palygorskite, the micromorphology of both pristine palygorskite and its hybrids was examined by scanning electron microscopy (Figure 1). Pristine palygorskite exhibits slender rod-like morphology with individual rod diameters of approximately 20–70 nm and lengths up to several micrometers. The rods are stacked in parallel or interwoven arrangements, leaving clearly visible gaps between them (Figure 1a). At low oxybenzone loadings (5% and 20%, Figure 1b,c), the rod-like structure is largely preserved, and the rods remain well-dispersed with no significant change in their apparent length or diameter. The inter-rod gaps are still observable, indicating that oxybenzone molecules are primarily adsorbed onto the external surface or incorporated into the internal spaces without causing visible morphological alterations. As the loading increases to 35% (Figure 1d), the gaps between rods begin to diminish, and the edges of the rods become slightly rounded. Some rod ends appear to be embedded in a continuous matrix, suggesting that oxybenzone has started to fill the inter-rod spaces. At the highest loading of 50% (Figure 1e), the gaps are nearly completely filled, and individual rods are no longer clearly distinguishable; instead, a compact, rounded aggregate morphology is observed. The rod edges are markedly rounded, and the surface appears smoother, which is attributed to the molten oxybenzone infiltrating the inter-rod spaces during the grinding and heating process, followed by recrystallization. This progressive change from well-dispersed individual rods to dense, rounded aggregates with increasing oxybenzone loading supports the interpretation that oxybenzone molecules are not only adsorbed onto the surface but also physically trapped in the inter-rod pores at higher loadings.

### 2.2. X-Ray Crystal Structure Analysis

**Figure 2:**
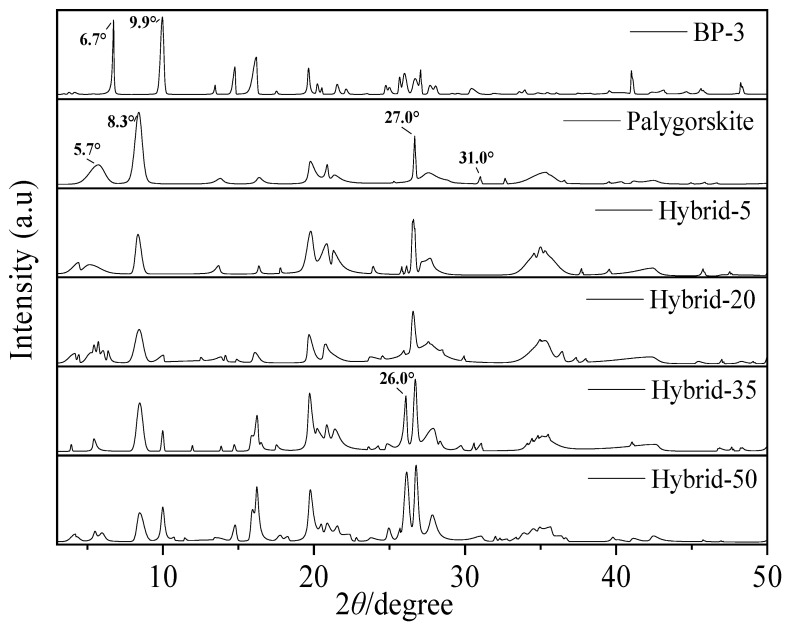
XRD patterns of oxybenzone, palygorskite and hybrids.

Oxybenzone, palygorskite, and their hybrid materials were characterized by X-ray diffraction (XRD). As shown in Figure 2, oxybenzone exhibited a well-crystallized diffraction pattern, with its strongest diffraction peak at 9.9° and a secondary strong peak at 6.7°. Palygorskite displayed the typical diffraction features of a chain-layered silicate mineral, where the main peak at 8.3° was attributed to the (110) crystal plane [26,32]. Furthermore, diffraction peaks corresponding to quartz (27.0°), montmorillonite (5.7°), and dolomite (31.0°) were detected in the raw material, which is consistent with the natural mineralogical attributes of palygorskite and indicates the presence of common associated impurities [33].

Significant changes were observed in the XRD patterns after hybridization. At low loadings (5% and 20%), all characteristic peaks of oxybenzone completely disappeared, and the intensity of the characteristic peaks of palygorskite itself decreased markedly. This phenomenon can be further explained by molecular size analysis. The minimum cross-sectional dimensions of the oxybenzone molecule are approximately 0.6–0.7 nm × 0.35 nm, which are larger than the theoretical nanochannel dimensions of palygorskite (0.37 nm × 0.64 nm) [34]. Therefore, the primary interaction mode of oxybenzone with palygorskite is not entry into the internal channels but rather adsorption onto the surface and within the inter-rod pores of palygorskite [35,36]. The preparation process of the hybrid disrupted the crystalline structure of oxybenzone, significantly disturbing its electron density distribution and leading to a substantial weakening of its diffraction intensity [37]. As the loading increased to 35% and 50%, characteristic peaks of oxybenzone (e.g., at 9.9° and 26.0°) re-emerged in the hybrids. However, these peaks were shifted in position, and their intensity increased with higher loading, while the characteristic peaks of palygorskite remained suppressed. This indicates that excess oxybenzone molecules recrystallized in the confined spaces on the surface and within the pores of palygorskite, forming a new crystalline morphology. The lattice of this new form differs from that of the bulk crystal, generating new diffraction signals that cause the peak shifts [35]. The XRD results demonstrate that a strong interaction beyond simple physical mixing occurred between oxybenzone and palygorskite. The disappearance of oxybenzone diffraction peaks at low loadings indicates molecular-level dispersion, not simple physical mixing.

### 2.3. Infrared Spectrum Analysis

**Figure 3:**
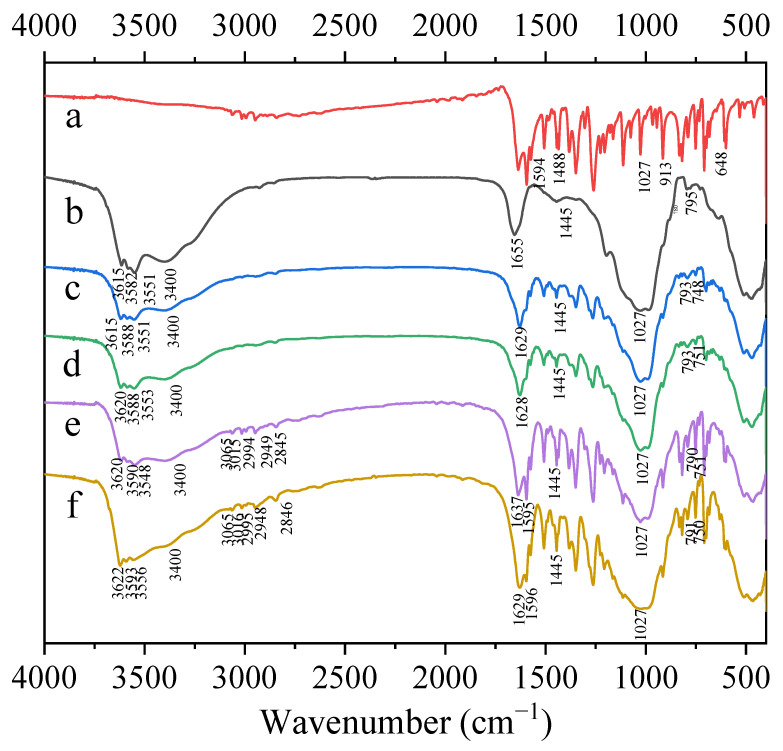
FTIR spectra of oxybenzone (**a**), palygorskite (**b**), hybrid-5 (**c**), hybrid-20 (**d**), hybrid-35 (**e**), and hybrid-50 (**f**).

Infrared spectroscopy is an effective technique for studying interactions between clay minerals and organic molecules [38]. Figure 3 displays the FTIR spectra of oxybenzone, palygorskite, and four hybrid materials. Oxybenzone exhibits distinct signals from its organic functional groups. The peaks at 3065, 3015, and 2994 cm^−1^ are attributed to aromatic C–H stretching vibrations, while those at 2948, 2888, and 2842 cm^−1^ originate from C–H stretching of alkyl chains (–CH_3_, –CH_2_–). The strong band at 1636 cm^−1^ is characteristic of the ketone carbonyl (C=O) stretching vibration, serving as a core feature of oxybenzone [39]. A series of medium-intensity bands in the region of 1594–1488 cm^−1^ correspond to aromatic ring skeleton (C=C) stretching vibrations. The bands between 1226 and 1075 cm^−1^ are assignable to methoxy group (C–O–C) stretching. The dense absorption peaks in the range of 900–650 cm^−1^ are characteristic of aromatic C–H out-of-plane bending vibrations.

For palygorskite, the band at 1445 cm^−1^ belongs to carbonate mineral impurities, and the peaks at 795 and 780 cm^−1^ are from quartz, indicating the presence of minor impurities in the raw material, which is consistent with the previous XRD results. In the high-frequency region, the bands at 3615 and 3581 cm^−1^ are attributed to the *v*(OH) stretching vibrations of Al_2_–OH, (Al,Fe)–OH, or (Al,Mg)–OH groups [40]. The band at 3551 cm^−1^ is associated with the *v*(OH) stretching of structural water. The broad band around 3400 cm^−1^ is attributed to physically adsorbed water, and the band at 1655 cm^−1^ represents the bending vibration of all O–H groups mentioned above.

When palygorskite forms hybrids with oxybenzone, the band at 1655 cm^−1^ disappears. This is associated with the organic molecules occupying the original sites of water molecules or disrupting their hydrogen-bonding network by interacting with hydroxyl groups or cation sites on the clay surface [41]. At low oxybenzone loadings (5% and 20%), the characteristic carbonyl band of oxybenzone at 1636 cm^−1^ also disappears and shifts to 1628 cm^−1^. This red shift is a typical indicator of the formation of C=O···H–O type hydrogen bonds, providing strong evidence for the interaction between the carbonyl groups of oxybenzone and the surface hydroxyl groups or adsorbed water of palygorskite. Notably, the bands in the structural hydroxyl region of palygorskite (3615–3551 cm^−1^) persist but undergo varying degrees of blue shift. This suggests that oxybenzone molecules interact with the M–OH groups on the palygorskite surface through relatively strong interactions, such as hydrogen bonding. This interaction alters the electronic environment or force constant of the hydroxyl groups in palygorskite to some extent, resulting in the frequency shift. The continued presence of the adsorbed water band around 3400 cm^−1^ indicates that the hybrid materials retain a certain degree of surface hydrophilicity.

As the oxybenzone loading increases further, the C–H stretching vibration bands of the aromatic ring and alkyl chains of oxybenzone (~3065, ~2948 cm^−1^, etc.) become clearly visible and their intensity increases. When the loading reaches 50%, the carbonyl vibration band of oxybenzone shifts from 1636 cm^−1^ to 1629 cm^−1^. This may be due to the tendency of the large number of oxybenzone molecules to form a dense, partially ordered coverage on the palygorskite surface. The high coverage forces nearly all oxybenzone molecules to stabilize primarily through hydrogen bonding between C=O and the surface. Despite the presence of other intermolecular forces, hydrogen bonding re-emerges as the dominant interaction, resulting in a significant and stable red shift of the C=O band. It is noteworthy that the Si–O stretching vibration band of the palygorskite framework (~1027 cm^−1^) remains stable in all hybrids [42], indicating that the basic crystal structure of palygorskite is not disrupted by the interaction. The FTIR analysis demonstrates that intermolecular interactions, predominantly hydrogen bonding, occur between oxybenzone and palygorskite via the melt-impregnation method. The red shift of the C=O band is indicative of hydrogen bonding, which is a chemical interaction beyond physical mixing. While additional techniques (e.g., solid-state NMR) could provide further confirmation, the current XRD and FTIR data strongly support the formation of a true hybrid structure.

### 2.4. Adhesion Effect of Modified Hybrid on Hair

To investigate the sun protection efficacy of the aforementioned hybrids, hair was selected as the target substrate, and the liquid-phase deposition method was initially intended for attaching the hybrids onto the hair surface. However, we regrettably found that all hybrids completely failed to adhere to the hair surface across the pH range of 4 to 10. The primary reasons for this failure are the poor interfacial compatibility between the hybrids and the hair, as well as the lack of an effective adhesion mechanism [43]. Consequently, surface modification of the hybrids is necessary to enhance their interaction with hair.

To improve the deposition outcome, two surfactants, polyquaternium-7 and polyvinylpyrrolidone, were introduced to modify the hybrids. These belong to cationic and non-ionic surfactants, respectively, and are widely used in cosmetic and personal care products [44]. Taking hybrid-20 as an example, after treatment with PQ-7, its FTIR spectrum exhibited new, strong peaks at 2929 and 2854 cm^−1^, corresponding to the alkyl chain C–H stretching vibrations characteristic of PQ-7. Following PVP treatment, characteristic peaks attributed to the amide group of PVP appeared at 1663 cm^−1^ (C=O stretching) and 1283 cm^−1^ (C–N stretching) (Figure S1). The emergence of these new peaks confirms the successful modification of the hybrid surface by both PQ-7 and PVP. The purpose of surface modification was to introduce functional groups that interact with hair keratin. PQ-7 (cationic) was chosen to provide electrostatic attraction to negatively charged hair at alkaline pH, while PVP (non-ionic) was chosen to provide hydrogen bonding with protonated hair groups at acidic pH. Successful modification was confirmed by FTIR: new peaks at 2929/2854 cm^−1^ (C–H stretching of PQ-7) and at 1663/1283 cm^−1^ (amide C=O and C–N of PVP) appeared after treatment (Figure S1).

**Figure 4:**
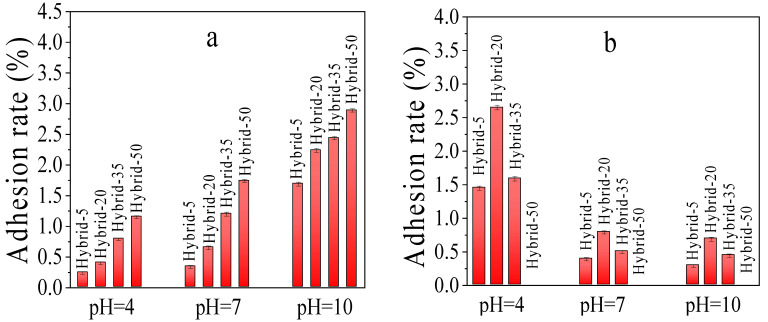
Adhesion rate of modified hybrids on hair at different pH ((**a**). polyquaternium-7, (**b**). polyvinylpyrrolidone).

The experimental results for hair deposition of the modified hybrid materials (Figure 4) indicate that the deposition rate of PQ-7-modified hybrids onto hair increased with both higher oxybenzone loading and higher pH values (Figure 4a). In contrast, for PVP-modified hybrids, the maximum deposition rate was observed at an oxybenzone loading of 20%. Furthermore, their deposition rate decreased significantly with increasing pH. When the oxybenzone content reached 50%, deposition onto hair failed completely for the PVP-modified hybrids (Figure 4b).

### 2.5. Adhesion Mechanism

#### 2.5.1. Colloidal Stability

The dispersion stability of nanoparticles in liquid phases significantly influences their deposition onto hair [26]. To elucidate the deposition mechanism of the aforementioned modified hybrid materials, we first investigated the kinetic stability of the hybrid colloidal systems by measuring the change in suspension transmittance over time (Figure S2). As shown in the figure, when the oxybenzone content was 5%, the colloidal stability slightly decreased compared to that of pure palygorskite. With a further increase in oxybenzone content, the colloidal stability gradually improved, reaching an optimum at 35%, and then declined to its poorest level at 50%. Simultaneously, for all hybrids, their stability generally exhibited an initial increase followed by a decrease with rising pH (Figure S2). After organic modification, the stability of all hybrids showed a trend of first increasing and then decreasing with increasing loading, with the best stability observed at 20% loading. This pH-dependent variation in colloidal stability can be interpreted as a reversible network formation, where the hybrid particles undergo network formation and disruption depending on surface charge and hydrophilicity. Furthermore, as can be seen from Figure S3, for the PQ-7-modified hybrids with loadings ≤ 35%, the colloidal stability of the three modified hybrids was similar. However, the difference between them gradually increased as the pH value rose. Except for hybrid-50, the suspension stability of the other samples gradually deteriorated with increasing pH. The PVP-modified hybrids exhibited a similar pattern. Specifically, the suspension stability was comparable between the hybrids with 5% and 20% loadings, and between those with 35% and 50% loadings, but the differences gradually widened with increasing pH. Except for hybrid-35, the suspension stability of the other hybrids showed a pattern of remaining stable initially and then deteriorating with changing pH (Figure S4).

Under acidic conditions, the suspension stability of hybrids modified with both surfactants was superior to that of the unmodified samples, except for hybrid-35. In a neutral environment, the modified hybrids exhibited better suspension stability than their unmodified counterparts, except for the PQ-7-modified hybrid with 35% loading. Alkaline conditions had a more pronounced negative impact on the suspension stability of modified hybrids with high loadings (≥35%) compared to the other modified hybrids. In contrast, for the PVP-modified hybrids under acidic conditions, the suspension stability was generally superior and showed less variation among different loadings. The PQ-7 modification system demonstrated a stronger stabilizing advantage for the sample with 20% loading under alkaline conditions. It is noteworthy, however, that regardless of surfactant treatment, the hybrid with 50% oxybenzone loading consistently exhibited the poorest suspension stability.

#### 2.5.2. Particle Size Analysis

**Figure 5:**
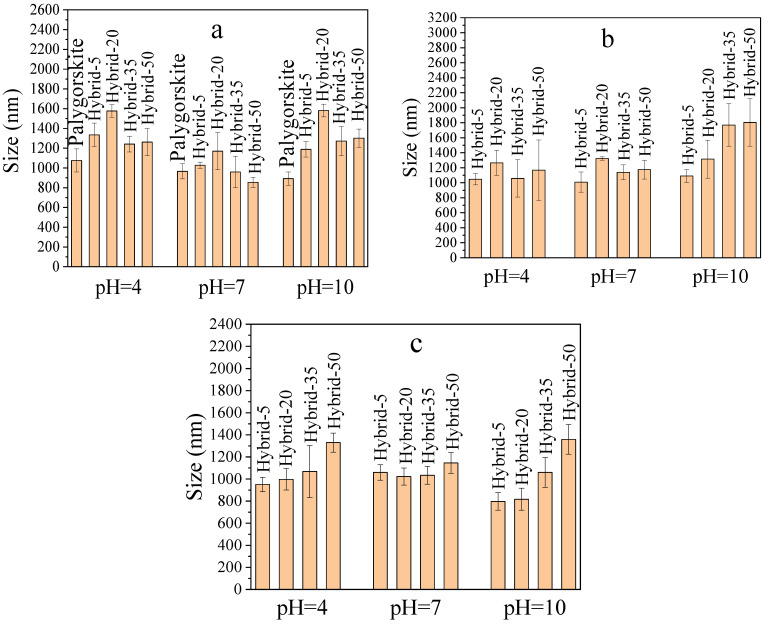
Particle sizes of palygorskite and hybrids (**a**), PQ-7-modified samples (**b**), and PVP-modified samples (**c**) at different pH.

The average particle size of the materials was first examined, and the results are presented in Figure 5. The average hydrodynamic diameter of all materials fell within the range between the nanometer and micrometer scales (approximately 800–1800 nm). Specifically, the particle size of palygorskite gradually decreased with increasing pH. The particle size of the hybridized samples was generally larger than that of pristine palygorskite and reached a minimum at pH 7. Across the entire pH range, the particle size of the hybrids first increased and then decreased with increasing oxybenzone content, peaking at a loading of 20%. After PQ-7 treatment, the particle size of hybrids with low loadings showed very limited variation across the entire pH range. In contrast, for samples with high loadings (≥35%), their particle size increased significantly in the alkaline environment (pH = 10); for example, the size of hybrid-50 increased from 1302 nm to 1803 nm. Conversely, after PVP treatment, the particle size of all hybrids increased with higher oxybenzone content, a phenomenon particularly pronounced under acidic or alkaline conditions. Smaller nanoparticles exhibit more intense Brownian motion and a lower tendency to settle in a gravitational field, indicating stronger kinetic stability [45]. Based on this, the PVP-modified samples generally demonstrated superior kinetic stability compared to the PQ-7-modified samples.

#### 2.5.3. Zeta Potential Analysis

**Figure 6:**
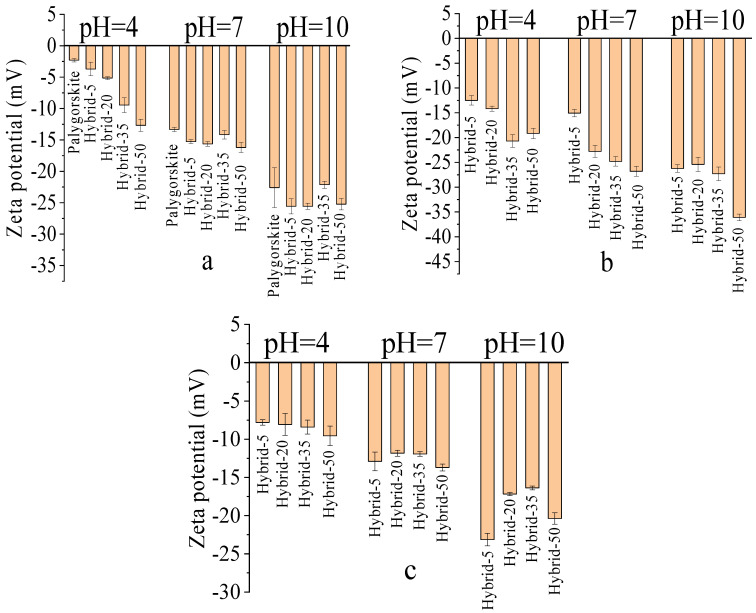
Zeta potentials of palygorskite and hybrids (**a**), PQ-7-modified samples (**b**), and PVP-modified samples (**c**) at different pH.

The surface charge of colloidal particles is another key factor influencing their dispersion stability. Sufficient electrostatic repulsion (typically corresponding to a zeta potential absolute value > 30 mV) can effectively prevent particle aggregation [46]. This study systematically measured the zeta potential of the hybrids and their surfactant-treated counterparts at different pH values, with the results shown in Figure 6. For the unmodified hybrids, their zeta potential decreased with increasing pH, which originates from the deprotonation of hydroxyl groups on the palygorskite surface [47]. This foundational strategy of utilizing the hydrophilic palygorskite carrier to improve the colloidal stability of the hydrophobic active ingredient has been confirmed as an effective approach by recent research [26]. Notably, as the oxybenzone loading increased from 5% to 50%, the zeta potential of the hybrids gradually decreased under acidic conditions, which can be attributed to the interaction between oxygen-containing groups (e.g., C=O) in benzophenone molecules and the palygorskite surface via coordination or hydrogen bonding. The exposed polar groups from the loaded molecules enhance the negative charge density at the interface. Especially under acidic conditions, partial deprotonation of certain functional groups (e.g., –COOH → –COO–) further reduces the net positive charge, thereby lowering the zeta potential. The changes under neutral and alkaline conditions were less pronounced than under acidic conditions, although the zeta potential was slightly higher for the hybrid with 35% loading compared to the others. This may be related to the enrichment of oxybenzone on the palygorskite surface, partially shielding its inherent negative charge sites.

The introduction of surfactants differentially modulated the surface charge of the materials. Specifically, after PQ-7 treatment, the zeta potential of the hybrids became more negative under neutral and alkaline conditions. In particular, hybrid-50 reached −36.1 mV at pH = 10, surpassing the classical stability threshold. This is attributed to the dense interfacial layer formed through multi-point adsorption of the long-chain PQ-7 polymer, creating a synergistic steric-electrostatic stabilization effect. This mechanism aligns with recent findings that surfactants/polymers as stabilizers can significantly extend the colloidal stability period of nanoparticles [48]. In contrast, PVP, as a non-ionic polymer, had a minimal effect on the zeta potential. The potential values of the treated samples were close to those of the untreated ones, and for some hybrids under identical conditions, the potential values were even higher than those of their unmodified counterparts, confirming its inability to effectively regulate surface charge.

#### 2.5.4. Contact Angle Analysis

The wettability of particle surfaces determines their dispersion stability in aqueous phases. Hydrophilic surfaces facilitate the formation of a robust hydration layer, which prevents particle aggregation through the steric hindrance effect [49]. Contact angle and immersion time were measured to evaluate the surface hydrophilicity/hydrophobicity of the materials, with the results presented in Table 1. Oxybenzone exhibited a contact angle of 85.7°, indicating very weak hydrophilicity. In contrast, palygorskite showed a contact angle of 26.9°, demonstrating strong hydrophilicity and making it an excellent hydrophilic nanocarrier [50]. Furthermore, when the oxybenzone loading increased from 5% to 50%, the contact angle of the hybrid materials increased from 50.8° to 60.8°, and the immersion time increased sharply from 8.4 s to 259.9 s. This is likely because at 5% loading, BP-3 molecules had already formed a nearly complete monolayer through surface enrichment, dominating the surface chemistry. Further increases in loading only thickened or patched this organic layer, leading to a diminishing return in enhancing the wettability of the outermost surface, thus resulting in a plateau in contact angle change. This observation directly correlates with the previously noted phenomenon of “poor dispersibility and a tendency to float at the air-water interface for hybrids with high loadings.”

The introduction of surfactants effectively modulated the interfacial wettability of the hybrids. Treatment with PQ-7 demonstrated an exceptional hydrophilic effect, significantly reducing the contact angles and drastically shortening the immersion times for all hybrids. Taking hybrid-35 as an example, its immersion time decreased from 106.1 s to 36.4 s after PQ-7 modification. This improvement is attributed to the oriented adsorption of PQ-7 molecules onto the particle surface, where the strongly hydratable cationic head groups form a stable hydrophilic interface. This strategy aligns with studies utilizing organic polymers to modify clays for tailoring their surface properties [51]. In comparison, polyvinylpyrrolidone was markedly less effective than polyquaternium-7 in enhancing the hydrophilicity of the hybrids. Notably, at high loadings (50%), PVP even reduced the hydrophilicity of the hybrids (increasing the contact angle from 60.8° to 67.9° and prolonging the immersion time to 1159.2 s), suggesting an adsorption limit for PVP on weakly hydrophilic interfaces.

Overall, among the four hybrid materials, hybrid-35 exhibited the best colloidal stability, which is attributed to the combined effect of its hydrophilicity/hydrophobicity balance and particle size. Although the particle size of hybrid-5 was smaller than that of hybrid-20, the latter showed slightly superior suspension stability due to its stronger surface electronegativity. While hybrid-50 had the smallest average particle size, its surface was the least hydrophilic among all hybrids, resulting in the poorest colloidal stability.

Both PQ-7-and PVP-modified hybrid-20 demonstrated the best colloidal stability within their respective modification systems, a result of surfactants improving the surface hydrophilicity and regulating the particle aggregation state of the hybrids. When treated with PQ-7, a uniform and dense hydrophilic layer could form effectively on hybrid-5 due to the good interaction between PQ-7 and the negatively charged sites on the palygorskite surface, leading to good colloidal stability. In contrast, for hybrid-35, the electrostatic binding sites were covered by oxybenzone, preventing the formation of an effective hydrophilic layer with PQ-7 and thus resulting in poorer colloidal stability compared to hybrid-5. Although hybrid-50 possessed the strongest surface electronegativity, its weakest hydrophilicity led to the poorest colloidal stability in this system. For PVP-modified hybrids, adsorption primarily relies on hydrogen bonding between the carbonyl groups (C=O) on the PVP chains and the surface hydroxyl groups of palygorskite, as well as hydrophobic interactions or van der Waals forces with hydrophobic structures like the benzene rings of oxybenzone molecules within the hybrid. For hybrid-35, these two types of interactions could work synergistically, providing more diverse and stronger anchoring points for PVP adsorption. Conversely, the surface of hybrid-5 contained fewer oxybenzone molecules, making its adsorption rely mainly on hydrogen bonding, which is less firm and results in suboptimal steric hindrance effects, thereby leading to poorer colloidal stability than hybrid-35. Furthermore, the PVP-modified hybrid-50 exhibited the weakest hydrophilicity and the longest immersion time, corresponding to the poorest colloidal stability among all PVP-modified hybrids. These improvements in colloidal stability and surface wettability are critical, as they directly influence the particles’ ability to interact with biological interfaces, including both the hair surface during application and algal cells in aquatic environments. From an environmental perspective, hybrids with balanced hydrophilicity (e.g., hybrid-35) exhibit optimal colloidal stability, which is desirable for uniform dispersion in water and efficient pollutant capture. Conversely, highly hydrophobic particles (hybrid-50) tend to float at the air-water interface, which may reduce water-phase bioavailability but also limit mass transfer for adsorption. These particle characteristics (size, charge, wettability) not only dictate adhesion to hair but also determine the hybrid’s ability to form a network for pollutant sequestration.

In summary, the adhesion rates of the two types of modified hybrids on hair are regulated by both the pH of the suspension and the inherent properties of the hybrids themselves, but the patterns and underlying mechanisms are distinctly different. For the PQ-7-modified samples, the highest adhesion rate was achieved under alkaline conditions (pH = 10). At this pH, the carboxyl groups on the hair keratin surface are deprotonated [52], increasing the negative charge density and generating strong electrostatic attraction with the cationic polyelectrolyte PQ-7. This process is equivalent to forming structured composite aggregates between the negatively charged palygorskite particles and the hair surface, consistent with the “dilution-induced deposition” mechanism of cationic polymers, which facilitates the directional anchoring and deposition of particles on hair [53]. Concurrently, as the oxybenzone content in the hybrids increased, the hydrophilicity of the material surface decreased, making it easier for the particles to approach and interact with the hydrophobic hair surface, thereby leading to a gradual increase in adhesion rate. For the non-ionic PVP-modified samples, the highest adhesion rate was observed under acidic conditions (pH = 4). At this pH, the amino groups of hair keratin are protonated and carry a positive charge, forming a dense hydrogen-bonding network with the lactam rings of PVP molecules [54]. Within this system, hybrid-20 showed the maximum adhesion rate. However, when the hydrophilicity became excessively weak (at 50% oxybenzone loading), PVP failed to mediate effective bridging interactions between the hybrid particles and the hair surface, due to its adsorption saturation on the weakly hydrophilic interface. Coupled with the worsened particle dispersibility, this resulted in zero adhesion rate.

It is worth noting that colloidal stability (optimal for hybrid-35) refers to uniform dispersion in water, favored by balanced hydrophilicity and zeta potential. In contrast, hair photoprotection depends on particle deposition (adhesion) onto hair. Despite its poor colloidal stability due to hydrophobicity, PQ-7-modified hybrid-50 exhibits the highest adhesion at pH 10 because the hydrophobic particle surface facilitates approach to the hydrophobic hair surface, and the cationic PQ-7 polymer provides strong electrostatic attraction to the negatively charged hair keratin at alkaline pH.

### 2.6. Sunscreen Effect of Hair

Hair is a natural polymeric fiber composed of the protein keratin, comprising the outermost cuticle layer, the middle cortex layer, and the innermost medulla layer [55]. It is formed from approximately 20 amino acids, including about 17.5% cysteine, which is present in both the surface cuticle scales and the cortex. Two hair samples exhibiting the highest adhesion rates (namely, hair treated with PQ-7-modified hybrid-50 at pH = 10 and hair treated with PVP-modified hybrid-20 at pH = 4) were selected for UV aging experiments alongside virgin hair.

**Figure 7:**
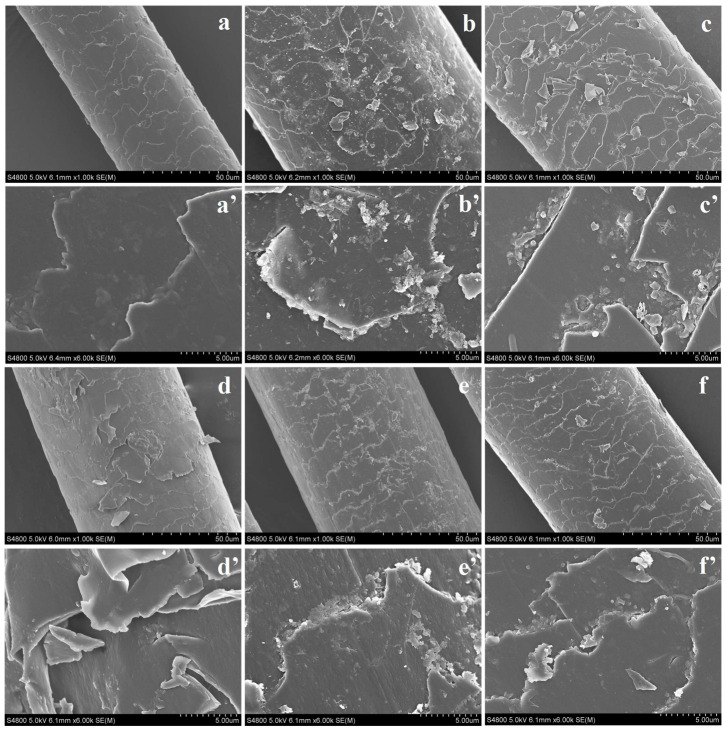
SEM photos of hair (**a**,**a’**), hair attached with hybrid-50 modified by polyquaternium-7 (**b**,**b’**), hybrid-20 modified by polyvinylpyrrolidone (**c**,**c’**) and the corresponding UV irradiated hair (**d**,**d’**,**e**,**e’**,**f**,**f’**).

Figure 7 shows scanning electron microscopy images of these hair samples before and after UV irradiation. The surface of virgin hair is clearly characterized by a regular distribution of numerous scale-like structures. The regions between the cuticle scales appear very smooth and flat, while their edges are thin and tightly overlapped, forming a uniformly oriented “stepped” texture (Figure 7a,a’). After the deposition of modified hybrids, particles are observed to be randomly distributed on the hair surface, with sizes ranging from several hundred nanometers to several micrometers (Figure 7b,b’,c,c’). The deposited particles self-assemble into a continuous protective coating layer that adheres tightly to the hair cuticle, providing effective UV shielding. This observation aligns with previously reported phenomena of nanoparticle adhesion onto hair [56,57]. Unlike the case with PVP-modified hybrid, a significant number of particles from the PQ-7-modified hybrid are found not only within the cuticle scale gaps but also adhered to the smooth epicuticle surface between the scales. Chemically, this epicuticle is primarily a covalently bonded lipid layer dominated by 18-methyleicosanoic acid [58]. This difference in deposition pattern can be attributed to the significantly weaker hydrophilicity of the PQ-7-modified hybrid-50 compared to the PVP-modified hybrid-20. Consequently, the former can more readily approach the hydrophobic epicuticle and become fixed.

After UV irradiation, significant damage is observed on the untreated hair, manifesting as partially lifted and even broken cuticle scales (Figure 7d,d’). In contrast, the two hair samples with adsorbed hybrids show almost no damage (Figure 7e,e’,f,f’), indicating the excellent UV-protective function provided by the hybrids. Furthermore, the UV-irradiated hair exhibits a distinct “photo-yellowing” phenomenon, characterized by an increased lightness (L* value) and a significantly elevated yellowness (b* value) in the colorimetric parameters. This is due to the partial degradation of melanin within the hair into small yellow molecular products upon UV exposure [59,60]. The changes in the colorimetric parameters for the two hair samples with adsorbed hybrids are markedly smaller than those for the untreated hair. A comparison of the color difference (Δ*E*) values confirms that the PQ-7-modified hybrid-50 provides the best sun protection performance (Table S1). This superior performance is likely related to its higher oxybenzone content, given that the adhesion rates of the two modified hybrids on hair were comparable (2.70% and 2.85%, respectively).

**Figure 8:**
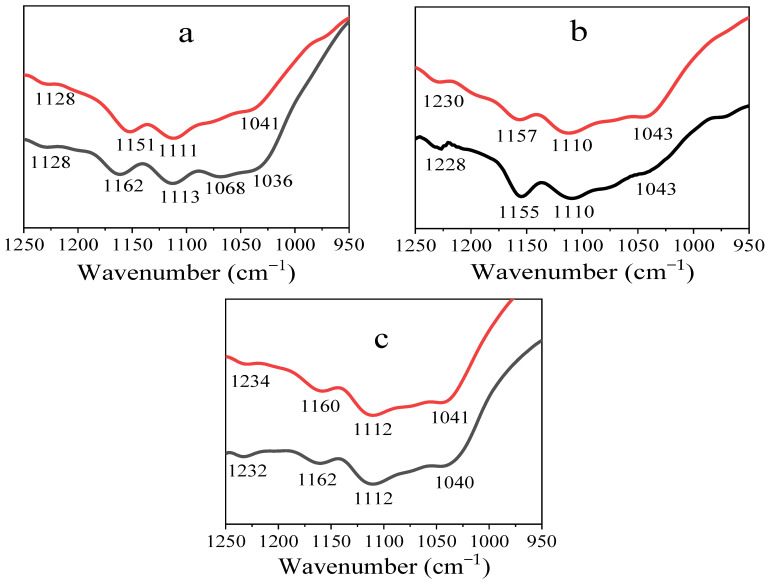
FTIR spectra of hair (**a**), hair attached with hybrid-50 modified by polyquaternium-7 (**b**), and hybrid-20 modified by polyvinylpyrrolidone (**c**) (The black and red lines represent the samples before and after UV irradiation, respectively).

To further understand the impact of ultraviolet light on the chemical composition of hair, FTIR spectra of the aforementioned hair samples were measured before and after irradiation (Figure 8). For untreated hair, characteristic absorption peaks attributed to the symmetric stretching vibration of the S=O bond in cysteic acid, a primary oxidation product of cysteine, were observed at 1068 cm^−1^ and 1036 cm^−1^ [61]. After UV exposure, the peak intensity at 1068 cm^−1^ significantly decreased, while the peak at 1036 cm^−1^ exhibited a red-shift, moving to 1041 cm^−1^. This phenomenon indicates a further alteration in the chemical environment of the initially formed cysteic acid or its precursors under sustained UV irradiation. The red-shift of the peak position is typically attributed to the enhancement of the hydrogen-bonding network surrounding the S=O bond or changes in the local microenvironment, which may result from the accumulation of oxidation products increasing the hydrophilicity of the protein or the formation of stronger intermolecular interactions with adjacent peptide chains [62]. Simultaneously, the spectral band assigned to the asymmetric stretching vibration of the S=O bond red-shifted from 1162 cm^−1^ to 1151 cm^−1^, providing further evidence for the systematic evolution in the chemical state of the sulfur-containing oxidation products. This synchronous and directional shift strongly suggests that the UV-induced oxidation process is not a random cleavage but follows a specific reaction pathway, potentially proceeding toward the formation of higher oxidation-state sulfur oxides (e.g., sulfoxides, sulfones) or causing local rearrangement of the protein’s secondary structure, thereby altering the vibrational energy levels of the chromophores [63]. It is particularly noteworthy that the absorption peak at 1128 cm^−1^, attributed to the stretching vibrations of single bonds such as C-O/C-N in the protein backbone, remained relatively stable before and after irradiation. This selective change underscores the chemical specificity of UV-induced damage to hair: the disulfide bonds (-S-S-) of cysteine and their oxidative derivatives are the primary sites of photochemical attack, exhibiting far greater sensitivity than the main-chain structure of the protein. For hair samples treated with the modified hybrid materials, no substantial changes were observed in the position or intensity of these characteristic peaks before and after UV irradiation, indicating that both types of modified hybrids provided effective protection against UV damage. The FTIR results are in complete agreement with the previously observed macroscopic phenomena of hair “photo-yellowing” (melanin degradation) and the physical cuticle damage observed under SEM, providing a coherent mechanistic explanation at the molecular level.

While direct UV–Vis transmission spectra of coated hair were not acquired, the UV-protective function is supported by three independent lines of evidence. First, SEM (Figure 7) shows that after UV irradiation untreated hair exhibits severe cuticle lifting and breakage, whereas hybrid-coated hair remains intact (Figure 7e,e’,f,f’). Second, colorimetry (Table S1) reveals a dramatic reduction in color difference Δ*E* from 10.51 (uncoated) to 1.60–2.29 (coated), indicating effective suppression of photo-yellowing. Third, FTIR (Figure 8) shows that the characteristic peaks of cysteic acid (oxidation product of cysteine) at 1068 cm^−1^ and 1036 cm^−1^ are significantly suppressed in coated hair, confirming protection at the molecular level. Direct UV–Vis transmission/absorption measurements on coated hair would provide quantitative SPF-like values and are planned for future studies. Nevertheless, the combination of morphological, colorimetric, and chemical evidence already provides strong support for the UV-protective performance of the deposited hybrids.

### 2.7. Effects of Hybrid on the Growth of Chlorella vulgaris

**Figure 9:**
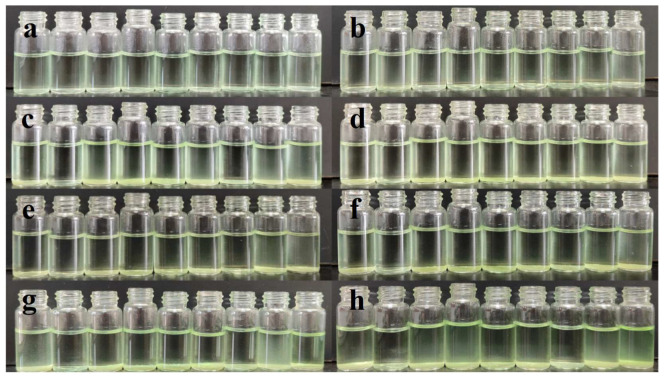
Photos of *Chlorella* growth ((**a**–**h**) is photos from 0 to 7 days, and each photo is blank, oxybenzone, hybrid-5, hybrid-20, hybrid-35, hybrid-50, polyquaternium-7, polyvinylpyrrolidone and palygorskite from left to right).

The hybrid particles adsorbed onto hair may detach due to human activities in coastal environments, potentially posing risks to aquatic organisms [64]. *Chlorella* is a spherical or oval-shaped unicellular alga, typically 3–8 μm in diameter, and generally grows and reproduces through photoautotrophy. It serves as an ideal model organism for studying the environmental impact of nanoparticles [1,65]. The effects of hybrid particles on the growth of *Chlorella* were investigated, and the results are shown in Figure 9. After one week of cultivation, the color of the *Chlorella* culture supplemented with oxybenzone became noticeably paler, indicating that it inhibited and killed a portion of the algal cells. This is attributed to its high toxicity to algae [66]. The color of the PQ-7 group remained largely unchanged, suggesting that it significantly inhibited the growth of *Chlorella*. In all other groups, the color gradually deepened with increasing cultivation time, with some groups even exhibiting a darker color than the blank control, indicating that the hybrids had a pronounced effect on the growth of *Chlorella*.

**Figure 10:**
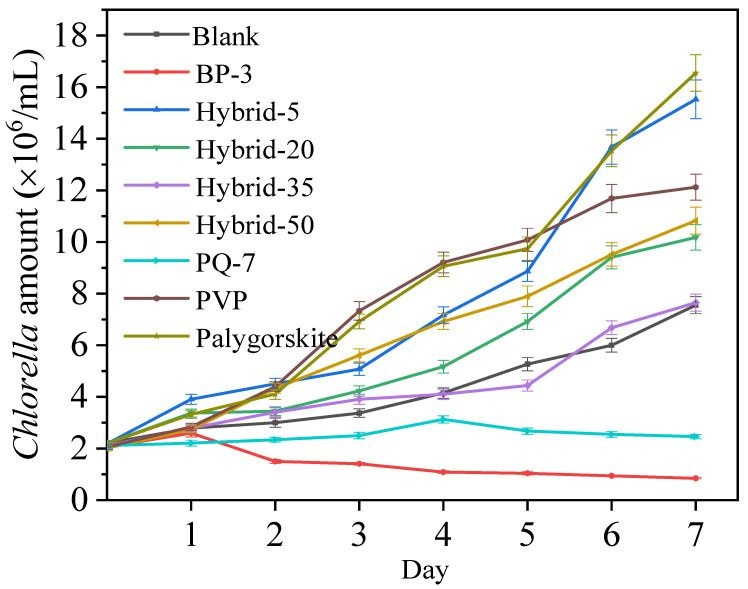
Effects of various substances on the growth of *Chlorella vulgaris*.

The effects of different additives on the growth of *Chlorella* are shown in Figure 10. In the blank control group, the number of *Chlorella* cells gradually increased over the cultivation period. In contrast, *Chlorella* cultivated with the addition of oxybenzone began to die on day 2, followed by a continuous decline in cell numbers. Conversely, *Chlorella* cultivated with palygorskite exhibited the best growth performance among all groups, with the final cell count exceeding twice that of the blank control. As the oxybenzone content in the hybrid materials increased, their growth-promoting effect on *Chlorella* gradually diminished. Notably, hybrid-50 showed the second strongest growth-promoting effect, following only hybrid-5.

The addition of PQ-7 resulted in significant growth inhibition of *Chlorella*, attributable to the bactericidal properties of this quaternary ammonium salt surfactant [67]. In comparison to the blank group, the addition of PVP promoted the growth of *Chlorella*. This aligns with recent findings on the significant growth-promoting effects (enhancing growth, nutrient removal, and product accumulation) of the non-ionic surfactant Tween 80 on *Chlorella* pyrenoidosa [68]. In essence, introducing the hybrid materials while maintaining an identical oxybenzone concentration promoted *Chlorella* growth.

According to particle size analysis (Section 2.5.2), the average diameter of both the pristine palygorskite and its hybrid materials was approximately 1 μm. Using the density of palygorskite, which is reported to be in the range of 2.05–2.32 g cm^−3^ [69], a value of 2.2 g cm^−3^ was adopted for calculation. It can be readily estimated that the number of these material particles in 100 mL of *Chlorella vulgaris* solution was approximately 5.56 × 10^8^ to 7.20 × 10^9^. Notably, this result is derived from a calculation based on a spherical model using the average particle diameter. Given that palygorskite crystals are inherently fibrous/rod-shaped, their actual volume is significantly smaller than that of an equivalent-diameter sphere. Consequently, the true particle number is likely to be more than an order of magnitude higher than this estimation. Therefore, the number of particles is much higher than that of *Chlorella* (about 200 million–1.6 billion in the whole culture period, Figure 7), which provides a great possibility for its full interaction with *Chlorella*.

Based on these cultivation results, we hypothesize the following mechanisms for palygorskite’s effect: Leveraging its large specific surface area and ion-exchange capacity, palygorskite can continuously adsorb and concentrate key macronutrients such as nitrogen (N) and phosphorus (P) from the culture medium. Furthermore, its fine particles can provide attachment sites for *Chlorella* cells. When palygorskite particles come into contact and adhere to algal cells in the aqueous phase, they can create a localized microenvironment enriched with nutrients around the cells. It is particularly noteworthy that positively charged metal elements present in the medium can neutralize the negative surface charges of both *Chlorella* and palygorskite, thereby reducing electrostatic repulsion and promoting mutual adhesion [70]. Additionally, palygorskite itself can adsorb metabolic byproducts from *Chlorella*, helping to purify the cultivation environment and create more favorable conditions. The particles may also act as condensation nuclei, promoting the flocculation of tiny *Chlorella* cells into larger aggregates [71]. While this may appear as sedimentation, it could potentially enhance material and informational exchange between algal cells, fostering a more favorable micro-ecosystem.

Consequently, it can be inferred that as the content of the toxic component (oxybenzone) in the hybrids increases, the binding sites on the palygorskite surface become covered, leading to a gradual reduction in its growth-promoting effect. However, when the oxybenzone proportion reaches 50%, the hydrophilicity of the hybrid material itself decreases significantly. This causes it preferential accumulation at the air-liquid interface, which effectively reduced its bioavailability to *Chlorella* cells in the aqueous phase and thus attenuated its inhibitory effect. This likely explains why its growth-promoting effect surpasses that of hybrid-20 and hybrid-35.

## 3. Conclusions

In this work, we successfully synthesized oxybenzone-loaded palygorskite nanohybrids with varying loadings (5–50%) via a melt impregnation method. The key findings and significance are summarized as follows.

(1) Hybrid formation and interactions: XRD and FTIR analyses confirmed strong hydrogen bonding between oxybenzone carbonyl groups and palygorskite surface hydroxyls, leading to stable organic–inorganic hybrids. The hydrophobic oxybenzone molecules are adsorbed onto the external surface and physically trapped in inter-rod pores, with excess loadings leading to recrystallization in confined spaces.
(2) pH-dependent adhesion mechanisms: While pristine hybrids show no affinity for hair, surface modification with cationic PQ-7 or non-ionic PVP enables effective deposition through two distinct, pH-controlled mechanisms. PQ-7-modified hybrids adhere optimally at pH 10 via electrostatic attraction to negatively charged hair keratin; PVP-modified hybrids adhere best at pH 4 via hydrogen bonding with protonated amino groups. The failure of PVP-modified hybrid-50 to adhere at high loading underscores the critical role of balanced surface hydrophilicity.
(3) Outstanding UV protection: The deposited hybrids significantly mitigate UV-induced hair damage, as evidenced by SEM (suppressed cuticle lifting), colorimetry (Δ*E* reduced from 10.51 to 1.60–2.29), and FTIR (inhibition of cysteine oxidation). The protective efficacy correlates with oxybenzone content and particle adhesion density.
(4) Environmental compatibility: Loading oxybenzone onto the palygorskite carrier dramatically reduces its ecotoxicity towards *Chlorella vulgaris*. Remarkably, pure palygorskite and low-loading hybrids promote algal growth, likely by providing nutrient-enriched microenvironments and attachment sites. This dual benefit of effective hair protection and reduced environmental risk highlights the potential of clay-based hybrids for sustainable personal care applications.

Overall, this work establishes a particle-property–function framework for designing functional hybrid materials with tailored surface properties. The findings provide fundamental insights into particle–biointerface interactions and offer a promising strategy for developing environmentally compatible sunscreens.

## 4. Materials and Methods

### 4.1. Materials

Palygorskite clay powder is provided by Anhui Mingguang Rare Mineral Co., Ltd. (Mingguang, China). According to the X-ray diffraction results (Figure 2), the mineral is mainly palygorskite, accompanied by a small amount of montmorillonite, quartz and dolomite. Human hair was sourced from Jinyi Hair Products Co., Ltd. (Heze, China) and trimmed into segments approximately 10 cm in length for subsequent use.

Oxybenzone (99%) was purchased from Damas-beta Reagent Company (Shanghai, China). Polyquaternium-7 was provided by Shanghai Ye Yuan Biological Technology Co., Ltd. (Shanghai, China). Polyvinylpyrrolidone (>98%) was obtained from Sinopharm Chemical Reagent Co., Ltd. (Shanghai, China). The stock culture of *Chlorella* species (cell density: 1.0 × 10^7^ cells mL^−1^) was sourced from Henan Huiduoli Biotechnology Co., Ltd. (Pingdingshan, China), and BG11 culture medium was supplied by Qingdao Hope Bio-Technology Co., Ltd. (Qingdao, China). Other reagents, such as hydrochloric acid and sodium hydroxide, were of analytical grade. Deionized water was used throughout the experiments.

### 4.2. Synthesis of the Oxybenzone–Palygorskite Hybrid

A series of hybrid materials with different ratios were prepared using the melt impregnation method. A measured amount of oxybenzone and palygorskite were manually ground in a mortar for 30 min to ensure sufficient mixing. Subsequently, the mixture was transferred into a conical flask with a stopper and placed in an oven, where it was heated from room temperature to 90 °C and maintained at this temperature for 12 h. The temperature of 90 °C was selected because it is slightly above the melting point of oxybenzone (62–64 °C), ensuring complete melting while avoiding thermal degradation. The heating time was set to 12 h (overnight) to guarantee sufficient interaction between oxybenzone and palygorskite. After natural cooling, the samples were collected for later use. The four hybrids were designated as hybrid-5, hybrid-20, hybrid-35, and hybrid-50, respectively, in accordance with the percentage content of oxybenzone in each hybrid.

### 4.3. Modification of Hybrid Materials

The hybrid materials were modified using polyvinylpyrrolidone and polyquaternium-7, respectively. Specifically, 1 g of the hybrid material was added to 100 mL of a 5 wt% surfactant solution. The mixture was subjected to high-speed dispersion at 15,000 rpm for 3 min using a T25 digital ULTRA-TURRAX^®^ disperser (IKA, Staufen, Germany) to achieve thorough dispersion. Subsequently, the suspension was transferred into a 250 mL conical flask and shaken at room temperature for 24 h. After shaking, the mixture was centrifuged at 10,000 rpm for 10 min by using H1850 high-speed centrifuge (Changsha Xiangyi Centrifuge Co., Ltd., Changsha, China), and the obtained solid was washed 3 times with deionized water. The final modified sample was dried to constant weight under vacuum for further use.

### 4.4. Stability Test of Suspension

Ten milligrams of the sample were added to 20 mL of deionized water and dispersed at 15,000 rpm for 3 min. The dispersion was then promptly transferred to a cuvette and placed in a 7200 UV spectrophotometer (UNICO (Shanghai) Instruments Co., Ltd., Shanghai, China). The change in transmittance over time was measured at a wavelength of 900 nm. The suspension stability of each sample was investigated at pH 4, 7, and 10.

### 4.5. Deposition of Hybrid Material on Hair

A total of 0.1 g of hybrid powder was added to 50 mL of water. The pH of the mixture was adjusted to a specific value using diluted HCl or NaOH, and then it was dispersed at 15,000 rpm for 3 min to form a suspension. Then, 0.1 g of hair was immersed in the suspension and incubated at room temperature (25 °C) for 150 min. After incubation, the hair was removed with tweezers, rinsed repeatedly with deionized water, and air-dried under ambient conditions. The adhesion rate of the hybrid material on hair was calculated by the weighing method using the following formula:

Adhesion rate (%) = (*m*_1_ − *m*_2_)/*m*_1_ × 100% (1)

where *m*_1_ and *m*_2_ represent the mass of the hair before and after deposition, respectively.

### 4.6. UV Accelerated Aging Test

In this study, a UV fluorescent aging test chamber (QUV/spray, Q-Lab Corporation, Westlake, OH, USA) was employed to conduct full-spectrum ultraviolet accelerated aging experiments to simulate solar ultraviolet radiation damage to hair. The apparatus was equipped with UVA-340 lamps, which closely match the solar spectrum in the 295–365 nm wavelength range and are specifically effective for ultraviolet degradation. The key operational parameters were set as follows: the irradiance at 340 nm was maintained at 0.68 W m^−2^, the chamber temperature during the irradiation period was set to 50 °C, and a continuous illumination mode was applied for 36 h. Prior to testing, a calibrated ultraviolet irradiance meter was used to perform multi-point measurements across the sample plane to ensure that irradiance fluctuations remained within ±10%. The treated hair strands (each weighing 0.1 g and of uniform length) were laid flat and secured on custom-made non-reflective aluminum sample holders to ensure uniform exposure.

### 4.7. Culture of Chlorella

To prepare *Chlorella* cultures with the desired initial cell density for experimentation, the following procedure was employed. A 20 mL aliquot of a stock culture with a cell density of 1.0 × 10^7^ cells mL^−1^ was accurately measured. Concurrently, BG11 medium was prepared by dissolving 0.08 g of the powdered formulation in deionized water, which was then brought to a final volume of 80 mL. This medium was sterilized by autoclaving at 121 °C for 15–20 min and allowed to cool to room temperature. Under aseptic conditions, the measured algal aliquot was combined with the sterilized medium. This resulted in a final culture volume of 100 mL with a target initial cell density of approximately 2.0 × 10^6^ cells mL^−1^. The prepared culture was subsequently incubated in a light incubator under the following conditions: a light intensity of 150 μmol·m^−2^·s^−1^, a photoperiod of 12 h light: 12 h dark, and a temperature of 25 °C.

The experiment was divided into nine groups. Group 1 served as the blank control. Group 2 Group 2 contained BP-3 at a concentration of 4 mg L^−1^. Groups 3 to 6 contained different concentrations of the hybrid materials, with the concentration of oxybenzone within these hybrids maintained at 4 mg L^−1^. Groups 7 and 8 contained PQ-7 and PVP, respectively, each at a concentration of 4 mg L^−1^. Group 9 contained palygorskite at a concentration equivalent to its content in the 5% hybrid formulation. All groups were cultured continuously for 7 days. The cultures were photographed daily, and the absorbance of the *Chlorella* suspension was measured at 680 nm. The quantity of *Chlorella* were calculated based on a standard curve (Figure S5) correlating the *Chlorella* amount with the absorbance (A). As palygorskite and its hybrids exhibited a baseline absorbance at this wavelength, its contribution was subtracted prior to calculating the actual *Chlorella* concentration.

### 4.8. Characterizations

The microscopic morphology of the samples was examined using a scanning electron microscope (SEM, Hitachi S-4800, Tokyo, Japan). Prior to imaging, the samples were sputter-coated with a gold film and observed under an accelerating voltage of 20 kV. X-ray diffraction (XRD) patterns were acquired on a Philips diffractometer (Philips, Amsterdam, The Netherlands) equipped with a Cu Kα radiation source (λ = 1.5418 Å), operated at 40 kV and 40 mA, with a scanning range from 3° to 50°. The zeta potential and particle size distribution of the hybrid materials were measured with a Malvern Zetasizer Nano ZS analyzer (Malvern Instruments Ltd., Malvern, UK), using the Zetasizer software (version 7.13, Malvern Panalytical Ltd.). Static water contact angles were determined using an OCA20 video-based optical contact angle measuring system (Dataphysics Instruments, Filderstadt, Germany) via the sessile drop method. The hybrid powders were first compressed into pellets (ca. 200 mg pressed at 10 MPa), and then the measurements were carried out on the pellet surfaces. Briefly, a 5 μL droplet of deionized water was deposited onto the material surface at 25 °C, and the contact angle was recorded after equilibration using the accompanying software. Each sample was measured in triplicate to quintuplicate, and the average value was reported. Fourier transform infrared (FTIR) spectroscopy was performed on a Thermo Nicolet NEXUS TM spectrophotometer (Thermo Fisher Scientific, Waltham, MA, USA) employing the KBr pellet technique. Spectra were obtained by averaging 32 scans with background subtraction. For hair samples, they were cut into approximately 1 mm segments prior to analysis. All spectra were collected within the range of 4000 to 400 cm^−1^ at a resolution of 4 cm^−1^. The color characteristics of the samples were determined in terms of CIE 1976 L*a*b* with an NH310 3nh High-Quality Colorimeter (Shenzhen 3NH Technology Ltd. Co., China), carried with the illuminant (D65) and observer angle (10°). Each sample was tested three times and the average value was taken. The color difference (Δ*E*) is calculated using the formula, Δ*E* = [(*L*_2_* − *L*_1_*)^2^ + (*a*_2_* − *a*_1_*)^2^ + (*b*_2_* − *b*_1_*)^2^]^1/2^. All data are presented as the mean ± SD (*n* = 3 independent measurements). The figures were prepared using Origin 2018 (OriginLab Corporation, Northampton, MA, USA).

## Figures and Tables

**Table 1:** Water contact angles and immersion times of oxybenzone, palygorskite, hybrids and modified samples.

Sample	Contact Angle (°)	Immersion Time (s)
BP-3	85.7 ± 3.1	∞
Palygorskite	26.9 ± 0.9	1.2 ± 0.1
Hybrid-5	50.8 ± 1.4	8.4 ± 0.3
Hybrid-20	52.2 ± 2.5	19.2 ± 0.5
Hybrid-35	53.6 ± 2.4	106.1 ± 2.6
Hybrid-50	60.8 ± 2.7	259.9 ± 5.7
Hybrid-5 with PQ-7	33.6 ± 1.3	4.7 ± 0.1
Hybrid-20 with PQ-7	36.5 ± 1.2	17.4 ± 0.2
Hybrid-35 with PQ-7	50.1 ± 2.1	36.4 ± 1.1
Hybrid-50 with PQ-7	57.4 ± 1.9	60.8 ± 2.5
Hybrid-5 with PVP	43.8 ± 1.1	7.4 ± 0.2
Hybrid-20 with PVP	45.7 ± 1.5	9.1 ± 0.3
Hybrid-35 with PVP	49.3 ± 1.6	26.6 ± 0.8
Hybrid-50 with PVP	67.9 ± 3.2	1159.2 ± 22.4

## Data Availability

The data presented in this study are available on reasonable request from the corresponding author.

## Supplementary Materials

The following supporting information can be downloaded at: https://www.mdpi.com/article/10.3390/gels12060530/s1, Figure S1: FTIR spectra of hybrid-20 (a), it modified by PQ-7 (b), and PVP (c); Figure S2: Curves of transmittance vs. time of suspensions of palygorskite and hybrids; Figure S3: Curves of transmittance vs. time of suspensions of hybrids modified by PQ-7; Figure S4: Curves of transmittance vs. time of suspensions of hybrids modified by PVP; Figure S5: Standard curve of *Chlorella* amount and absorbance; Table S1: Colorimetric data of hair samples before and after UV irradiation.
