# Supplementary material for: Interfacial Engineering of Clay-Based Nanohybrids with pH-Responsive Network-like Behavior for Hair Photoprotection and Algal Growth Promotion
Source: Gels. 2026 Jun 12;12(6):530. doi: 10.3390/gels12060530 (PMC13299635; doi:10.3390/gels12060530)
Supplement: Supplementary file 1 [file gels-12-00530-s001.zip › gels-4351828-supplementary.pdf]

Interfacial Engineering of Clay-Based Nanohybrids with pH-Responsive Network-Like Behavior for Hair Photoprotection and Algal Growth Promotion

Hao Chen*, Yufan Song

School of Pharmaceutical and Chemical Engineering, Taizhou University, Shifu Road No. 1139, Taizhou 318000, Zhejiang, PR China

*Corresponding author. Tel.: +86-576-85137265; Fax: +86-576-85137182;

E-mail address: chenhao2212@sohu.com

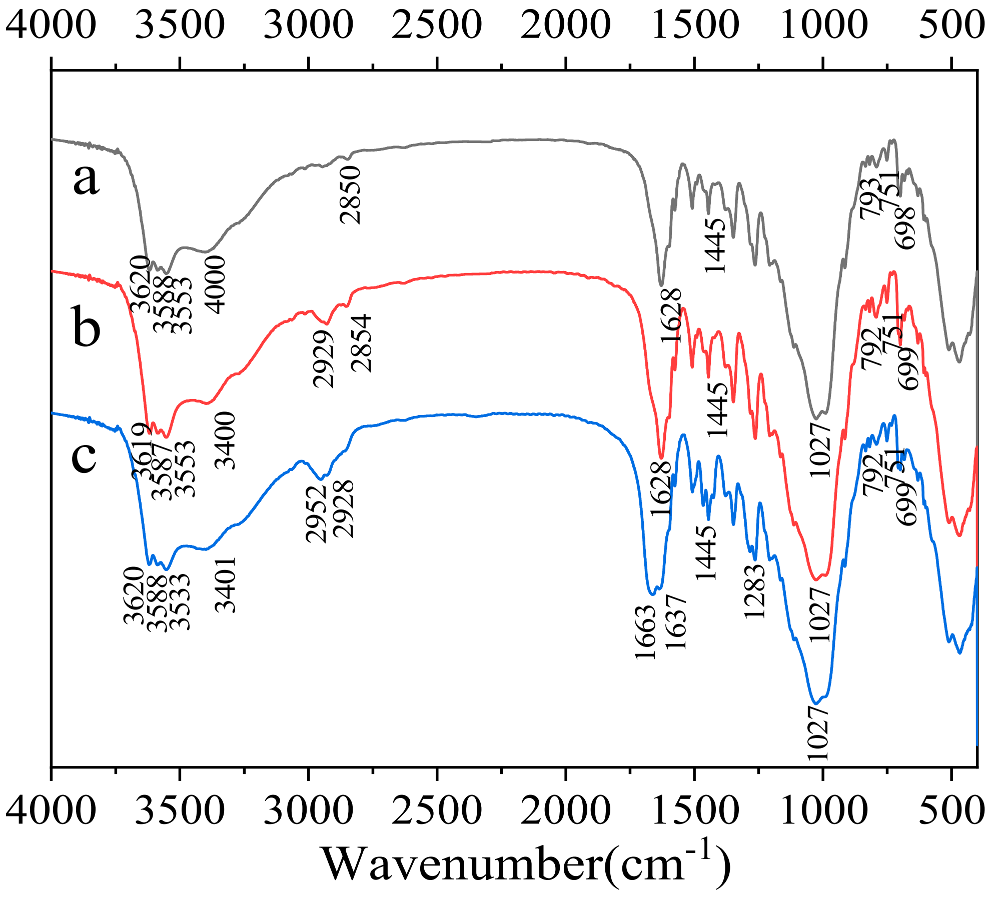

Figure S1. FTIR spectra of hybrid-20 (a), it modified by PQ-7 (b), and PVP (c).

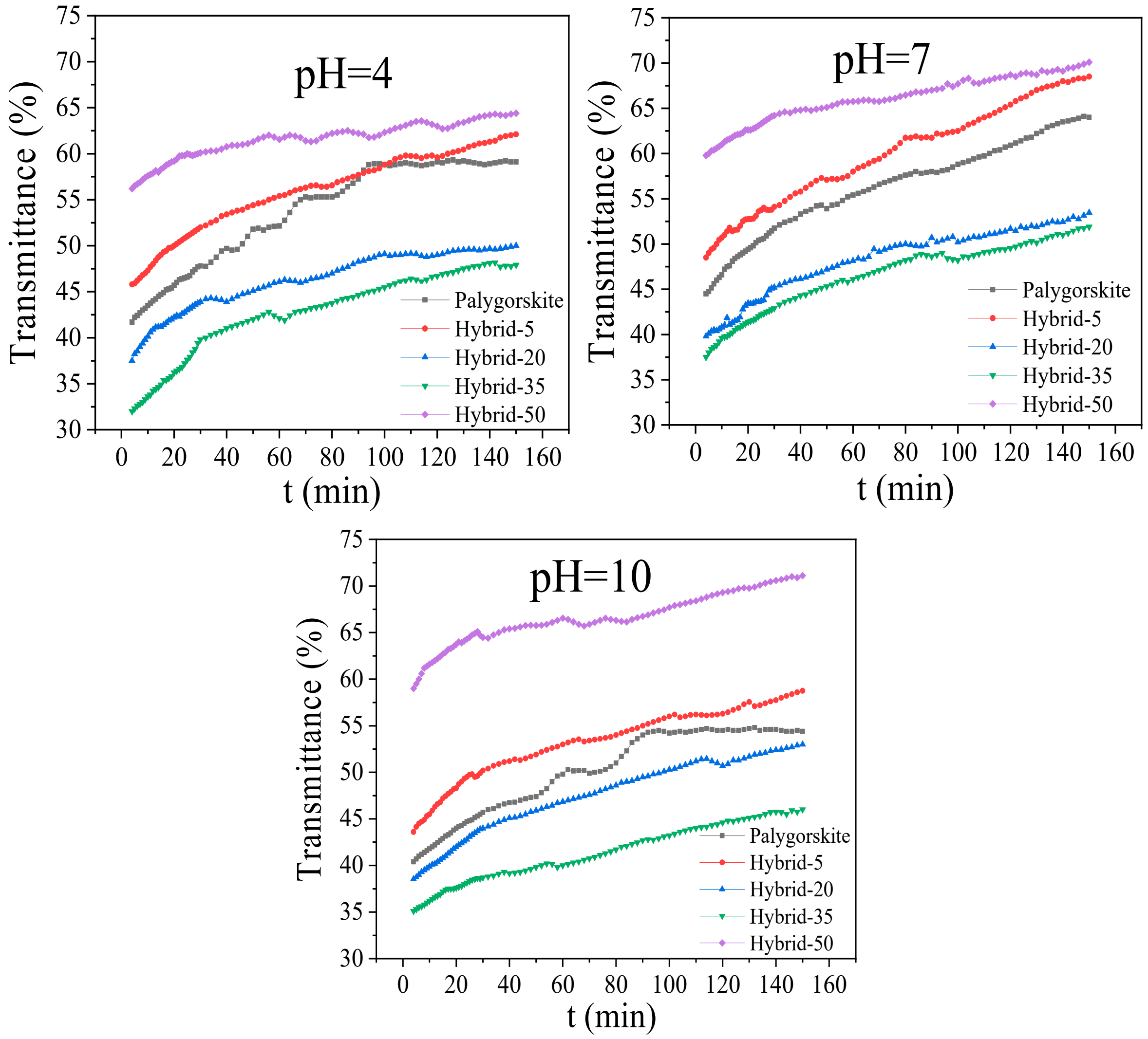

Figure S2. Curves of transmittance vs. time of suspensions of palygorskite and hybrids.

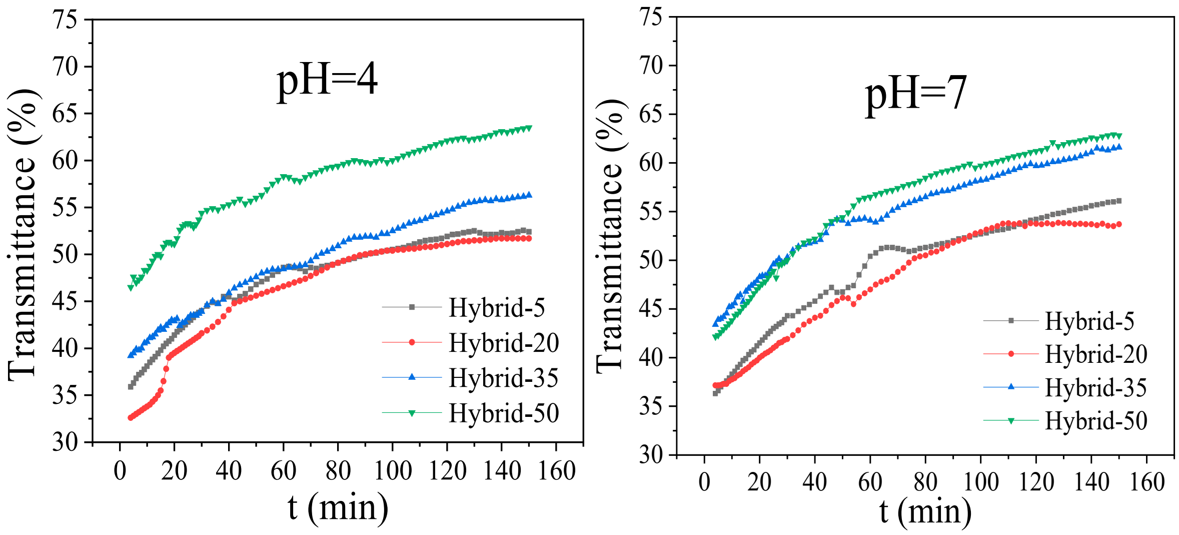

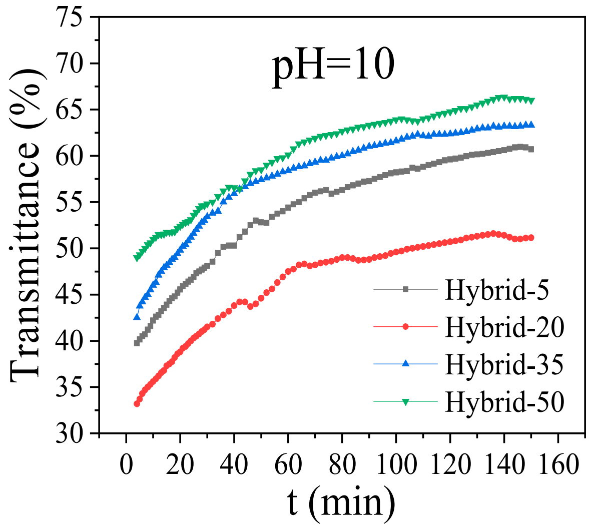

Figure S3. Curves of transmittance vs. time of suspensions of hybrids modified by PQ-7.

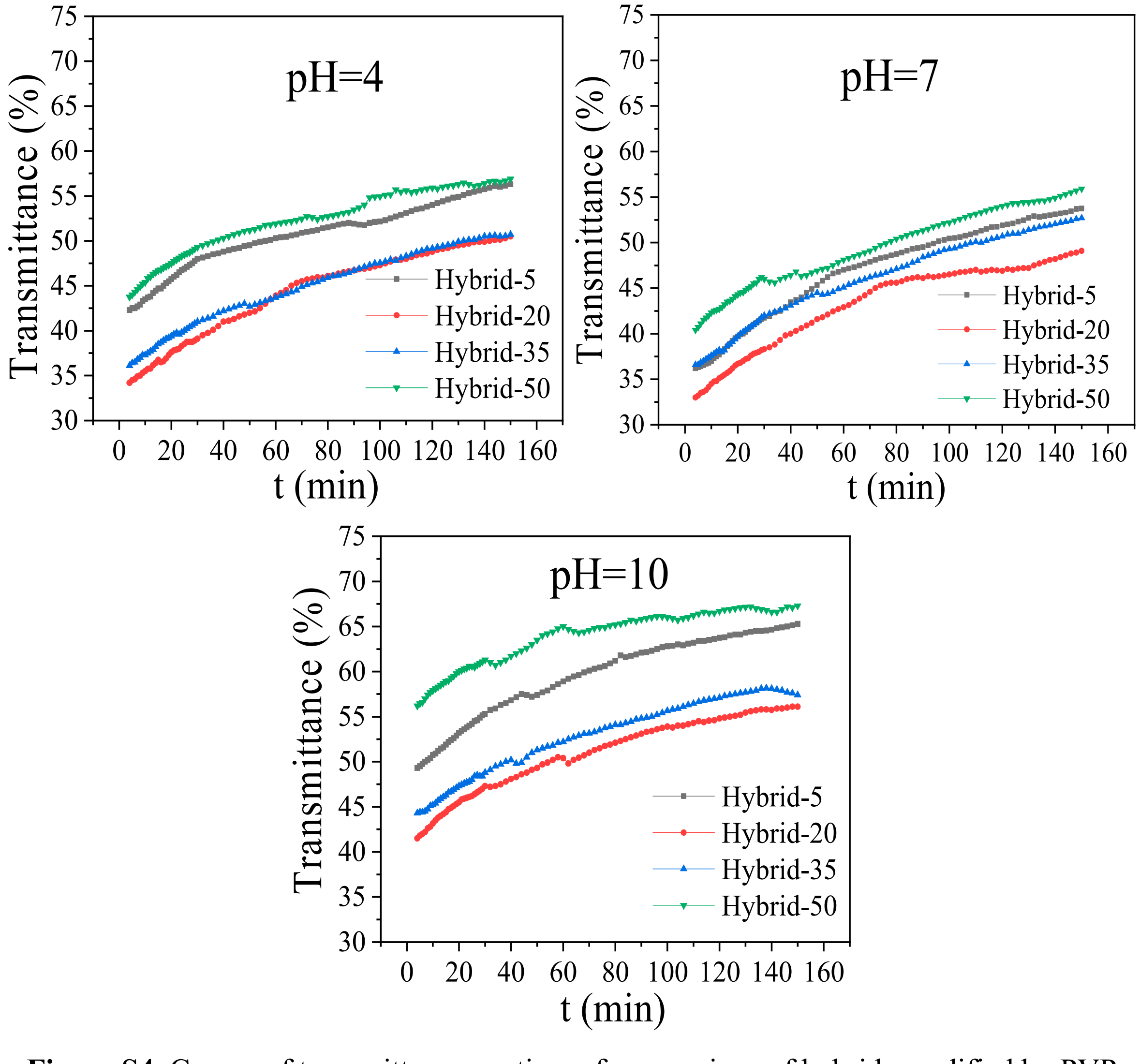

Figure S4. Curves of transmittance vs. time of suspensions of hybrids modified by PVP.

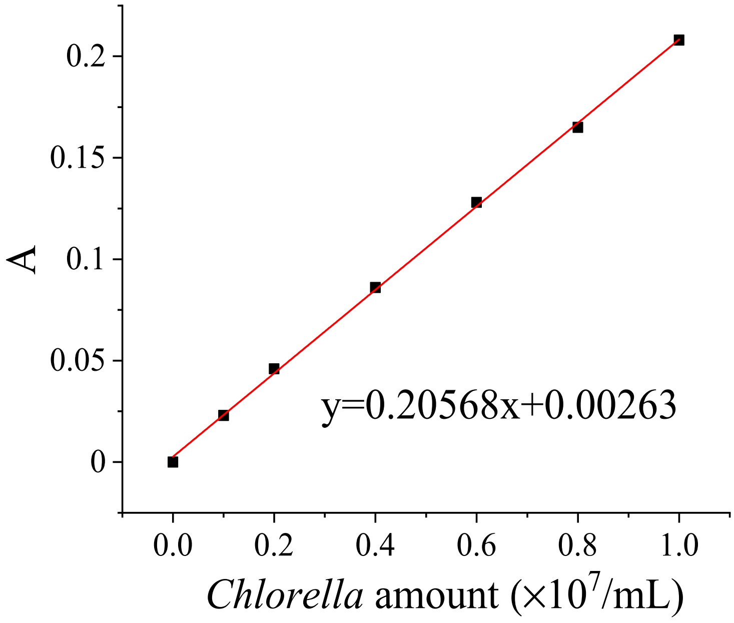

Figure S5. Standard curve of *Chlorella* amount and absorbance.

Table S1 Colorimetric data of hair samples before and after UV irradiation.

Sample	*L**	*a**	*b**	Δ*E*
Hair	20.85	1.52	0.88	
Hair after UV irradiation	28.47	1.33	8.12	10.51
Hair with PQ-7 modified hybrid-50	20.72	1.55	0.93	
Hair with PQ-7 modified hybrid-50 after UV irradiation	22.09	1.52	1.76	1.60
Hair with PVP modified hybrid-20	20.80	1.56	0.87	
Hair with PVP modified hybrid-20 after UV irradiation	22.55	1.50	2.34	2.29
